# Long Non-coding RNA HOTAIR in Central Nervous System Disorders: New Insights in Pathogenesis, Diagnosis, and Therapeutic Potential

**DOI:** 10.3389/fnmol.2022.949095

**Published:** 2022-06-23

**Authors:** Jialu Wang, Jiuhan Zhao, Pan Hu, Lianbo Gao, Shen Tian, Zhenwei He

**Affiliations:** ^1^Department of Neurology, The First Affiliated Hospital of China Medical University, Shenyang, China; ^2^Department of Neurology, The Fourth Affiliated Hospital of China Medical University, Shenyang, China

**Keywords:** long non-coding RNAs, central nervous system disorders, HOTAIR, pathogenesis, diagnostic value, therapeutic target

## Abstract

Central nervous system (CNS) disorders, such as ischemic stroke, neurodegenerative diseases, multiple sclerosis, traumatic brain injury, and corresponding neuropathological changes, often lead to death or long-term disability. Long non-coding RNA (lncRNA) is a class of non-coding RNA with a transcription length over 200 nt and transcriptional regulation. lncRNA is extensively involved in physiological and pathological processes through epigenetic, transcription, and post-transcriptional regulation. Further, dysregulated lncRNA is closely related to the occurrence and development of human diseases, including CNS disorders. HOX Transcript antisense RNA (HOTAIR) is the first discovered lncRNA with *trans-*transcriptional regulation. Recent studies have shown that HOTAIR may participate in the regulation of the occurrence and development of CNS disorders. In addition, HOTAIR has the potential to become a new biomarker for the diagnosis and prognosis assessment of CNS disorders and even provide a new therapeutic target for CNS disorders. Here, we reviewed the research results of HOTAIR in CNS disorders to provide new insights into the pathogenesis, diagnostic value, and therapeutic target potential of HOTAIR in human CNS disorders.

## Introduction

Central nervous system (CNS) disorders, including ischemic stroke, neurodegenerative diseases (NDDs), multiple sclerosis (MS), traumatic brain injury (TBI) and the subsequent neuropathic injuries, are the main causes of morbidity and mortality (Hong et al., 2019; Khellaf et al., 2019; Herpich and Rincon, 2020). However, due to the lack of beneficial treatments for these complex neuropathologies, CNS disorders are often accompanied by acute and chronic cellular damage (Shang et al., 2020; Troshev et al., 2021). Therefore, there is an urgent need for effective therapeutic methods to prevent secondary injury caused by treatment and to successfully treat CNS disorders (Marehbian et al., 2017; Esechie et al., 2019).

Long non-coding RNA (LncRNA) is a class of non-coding RNA with a transcription length over 200 nt and transcriptional regulation. Most lncRNAs are transcribed and spliced by RNA polymerase II, and some lncRNAs have both 5′ end caps and 3′ poly(A) tails (Derrien et al., 2012). Similar to protein-coding genes, some epigenetic modifications of lncRNAs are visible throughout the genome, but they usually have no functional open reading frame (Tan et al., 2021). Importantly, lncRNAs participate in the occurrence and development of many diseases (Riva et al., 2016; Bao et al., 2018). Accordingly, they can be used as a molecular sponge, modulator of signal pathways, epigenetic regulator, and molecular scaffold to extensively regulate various important biological activities, including cell proliferation, differentiation, growth and development, and cell apoptosis. Specifically, lncRNAs participate in various signal transduction regulation processes and play the following roles: (i) scaffold: binding to two or more proteins to play a regulatory role; (ii) decoy: inducing and combining with a series of regulatory factors to hinder its combination with the corresponding functional sites; (iii) guide: recruiting specific proteins and combining with them to form complexes; (iv) signal: lncRNA can reflect the regulation of genes by transcription factors or signal pathways in space and time (Wang and Chang, 2011).

lncRNAs interact with other biomolecules (e.g., DNA, RNA and proteins) and play an important role in biological processes through several mechanisms, such as acting as inhibitory sponges for microRNAs (miRNAs), participating in chromatin remodeling and affecting protein stability (Jalali et al., 2017). lncRNAs can be expressed in multiple tissues, but the highest expression levels are found in the CNS (Policarpo et al., 2021). Several lncRNAs have been shown to play important roles in the regulation of CNS development, and the ability of lncRNAs to participate in the regulation of hundreds of transcriptomes against CNS injury and disorder makes them candidates for stabilizing transcriptomic homeostasis and as promising therapeutic targets (Wu et al., 2013). HOTAIR is one of the most extensively studied lncRNAs found dysregulated in human tumors. Although it does not encode proteins, it is involved in RNA processing, gene regulation, chromatin modification, gene transcription, and post-transcriptional regulation.

HOTAIR expression level can well reflect the disease state and can be used as a potential biomarker (Liu et al., 2020). Studies have shown that HOTAIR can competitively inhibit some target miRNAs to act as its molecular sponge to release miRNA inhibition on target messenger RNA (mRNA) and play specific biological functions (Rajagopal et al., 2020; Chi et al., 2021). HOTAIR plays an indispensable role in many pathophysiological processes through epigenetic regulation (Tsai et al., 2010). Accordingly, HOTAIR has been extensively studied in various types of tumors, and results have shown that it is widely involved in tumor cell proliferation, apoptosis, angiogenesis, invasion, and metastasis (Gupta et al., 2010; Yang et al., 2018; Zhang J. et al., 2020). Moreover, emerging evidence confirms that HOTAIR is also widely involved in the pathogenesis of CNS disorders and can be a potential diagnostic marker and therapeutic target of CNS disorders. It has a broad clinical application prospect in the early diagnosis, efficacy judgment, prognosis prediction, and gene therapy of CNS disorders (Jin Z. L. et al., 2021; Momtazmanesh and Rezaei, 2021).

In this review, we outline the mechanisms by which HOTAIR exerts its regulatory function and summarize its role in the pathogenesis of ischemic stroke, NDDs, MS, and TBI. Finally, the potential of HOTAIR as a disease diagnostic biomarker of CNS disorders is highlighted.

## lncRNA HOTAIR

Human homeobox (HOX) is a 2,158-nt, single-strand gene transcribed in an antisense manner from the HOXC locus on chromosome 12q13.13, one of the chromosomal loci of the clustered HOX genes (HOXA, B, C, and D) (Gupta et al., 2010). HOTAIR was first discovered by Rinn et al. (2007) as a special lncRNA that regulates gene expression by *trans-*silencing chromatin. The human HOTAIR gene is located in the intergenic region between HOXC11 and HOXC12 in the HOXC cluster on chromosome 12 (Li et al., 2017). HOTAIR can recruit polyclonal repressor complex 2 (PRC2) at the 5′ end to inhibit the expression of homeobox gene cluster D (Hung and Chang, 2010). Histone modifications are essential for transcriptional activation, and PRC2 contains the enhancer of Zeste homolog 2 (EZH2), a histone methyltransferase that marks transcriptionally repressed genes by trimethylation at lysine 27 of histone H3 (H3K27me3) (Duan et al., 2020). HOTAIR binds to GA-rich motifs in the genome to form a broad domain occupied by PRC2 and subsequently H3K27me3 (Song et al., 2019).

**FIGURE 1:**
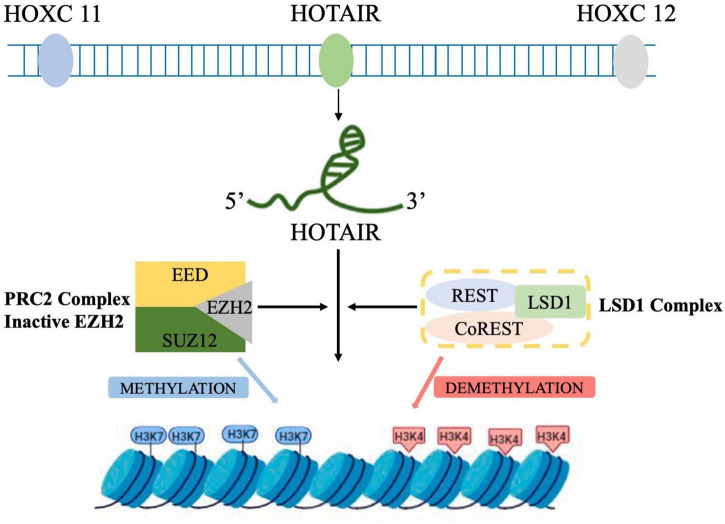
Schematic representation of HOTAIR-mediated gene silencing. HOTAIR acts as a molecular scaffold, bridging PRC2 and LSD1 complexes and altering gene expression by promoting H3K27me3 and H3K4 demethylation (by PRC2 and LDS1, respectively) at the target genes.

Lysine-specific demethylase 1 (LSD1), a member of the amine oxidase family, forms a complex with repressor element 1 silencing transcription factor (REST) corepressor 1 (CoREST1), which acts as a bridge to link LSD1 to REST to form LSD1/CoREST/REST complex. LSD1/CoREST/REST complex mediates histone H3 lysine-4 dimethylation (H3K4me2) demethylation to regulate the transcriptional activity of target genes. The 3′ end of HOTAIR binds to a compound containing LSD1 to inactivate gene expression *via* H3K4me2 (Figure 1). Overall, HOTAIR has specific bidirectional binding ability, that is, the 5′-end domain binds to the PRC2 complex, while its 3′-end domain binds to the LSD1/REST/CoREST complex. In addition, HOTAIR can act as a scaffold to guide PRC2, and LSD1 forms a complex and mediates the complex to a specific genomic locus to demethylate the chromosomes H3K27me3 and H3K4me2. Thus, the chromosome is maintained in a closed state maintaining, and the corresponding downstream genes are silenced (Tsai et al., 2010).

## Mechanisms of HOTAIR Function

HOTAIR plays an important role in the development of CNS disorders (Table 1). It is closely associated with inflammatory response, cell apoptosis, oxidative stress, and autophagy. However, the relevant molecular mechanisms are still unclear. Here, we summarize the molecular mechanisms related to the function of HOTAIR.

**TABLE 1:** Role of HOTAIR in CNS disorders.

Neurological disorders	Animal models/Cell employed	Regulation	Target genes/Pathway	Potential therapeutic effect	References
Ischemic stroke	pMCAO C57BL/6 mice/OGD-injured N2a	Upregulated	KLF6/miR-148a-3p/KLF6 axis and STAT3 pathway	Inhibit neural cell inflammatory response and apoptosis, increase cell viability, decrease infarcted area and neurological deficits.	Huang et al., 2021
	OGD/R-induced hBMVECs	Upregulated	EZH2, Bax, caspase-3, Bcl-2, occludin, claudin-5, ZO-1, VE-cadherin	Maintain BBB permeability and anti-apoptosis	Wang et al., 2022a
	pMCAO ICR mice/hypoxia induced HT22 cells	Upregulated	NOX2	Inhibit cell apoptosis	Yang and Lu, 2016
AD	HeLa cells/brain tissue samples of AD patients	Downregulated	CDK5R1/miR-15/107	Reduce Aβ production and Tau protein hyperphosphorylation	Spreafico et al., 2018
	APP/PS1 mice	Upregulated	miR-130a-3p	Anti-inflammation	Lu et al., 2022
	Sevoflurane-mediated SD rats	Upregulated	*Bdnf*	Increase BDNF expression in hippocampus	Wang J. Y. et al., 2018
	ISO-evoked SD rats/HT22 cells	Upregulated	miR-129-5p	Reverse the injury of ISO on cell viability, inflammation, apoptosis, and oxidative stress	Wang et al., 2022b
PD	MPTP induced C57BL/6J mice/MPP^+^ induced SH-SY5Y	Upregulated	miR-221-3p/α-synuclein	Increase cell viability, reduce cell apoptosis, inhibit inflammatory cytokines secretion and oxidative stress reaction	Sun et al., 2022
	MPP^+^ induced SK-N-SH cells	Upregulated	miR-874-5p/ATG10	Anti-autophagy	Zhao et al., 2020
	MPTP induced C57BL/6J mice/MPP^+^ induced SH-SY5Y	Upregulated	miR-326	Inhibit NLRP3 mediated pyroptosis activation	Zhang Q. et al., 2021
	MPTP induced C57BL/6J mice/MPP^+^ induced SH-SY5Y	Upregulated	miR-126-5p/RAB3IP	Inhibit autophagy and cell apoptosis	Lin et al., 2019
	MPTP-induced C57BL/6/MPP^+^-induced MN9D	Upregulated	miR-221-3p/NPTX2	Inhibit autophagy in the substantia nigra compacta	Lang et al., 2020
	MPTP-induced C57BL/6/MPP^+^-induced MN9D	Upregulated	SSTR1	Inhibit dopaminergic neuron apoptosis	Cai et al., 2020
	MPTP induced C57BL/6 mice/MPP^+^ induced SH-SY5Y	Upregulated	LRRK2	Inhibit dopamine neuronal apoptosis by suppressing caspase 3 activity	Wang S. et al., 2017
	MPTP induced C57BL/6 mice/MPP^+^ induced SH-SY5Y	Upregulated	LRRK2	Anti-apoptosis	Liu S. et al., 2016
MS	MOG_35–55_ induced C57BL/6 mice	Upregulated (VD deficiency)	NA	Immunomodulatory effects	Pahlevan Kakhki et al., 2018
	Cuprizone induced C57BL/6 mice/	Upregulated	miR-136-5p	Inhibit microglia activation promote myelin regeneration	Duan et al., 2018
TBI	Craniotomy C57BL/6 mice	Upregulated	MYD88	Inhibit microglia overactivation and inflammatory factor release after TBI	Cheng et al., 2021

*AD, Alzheimer’s disease; ATG10, autophagy-related 10; BBB, blood-brain barrier; Bdnf, brain-derived neurotrophic factor; CDK5R1, cyclin-dependent kinase 5 regulatory subunit 1; EZH2, enhancer of Zeste homolog 2; hBMVECs, human brain microvascular endothelial cells; ISO, isoflurane; LRRK2, leucine-rich repeat kinase 2; MOG, myelin oligodendrocyte glycoprotein; MS, multiple sclerosis; MYD88, myeloid differentiation factor-88 adaptor protein; NLRP3, NOD-like receptor (NLR) family pyrin domain-containing protein 3; NOX2, nicotinamide adenine dinucleotide phosphate oxidase oxidases 2; NPTX2, neuronal pentraxin II; OGD, Oxygen–glucose deprivation; PD, Parkinson’s disease; pMCAO, permanent middle cerebral artery occlusion; RAB3IP, Rab3a interacting protein; SD, Sprague–Dawley; SSTR1, somatostatin receptor 1; TBI, Traumatic Brain Injury; VE, vascular endothelial; ZO-1, zonula occludens-1.*

### AS Competitive Endogenous RNA

Competing endogenous RNAs (ceRNA) hypothesis reveals a novel mechanism of RNA interaction. MiRNAs are known to cause gene silencing by binding mRNAs, while ceRNA can competitively bind miRNAs to regulate gene expression (Thomson and Dinger, 2016). ceRNA can bind to miRNAs through miRNA response elements (MREs) to affect miRNA-induced gene silencing. Recent studies have shown that ceRNA is an important mechanism for the occurrence and development of various CNS disorders (Moreno-García et al., 2020; Luo et al., 2021). Interaction with target sites in the mRNA 3′ UTR can lead to reduced mRNA stability and translational repression, thereby regulating RNA gene expression (Amort et al., 2013). Several studies have shown that HOTAIR is an important ceRNA that mainly serves as a miRNA sponge in the body (Xu et al., 2016). In breast cancer (BC), HOTAIR upregulates HMGA2 expression by competitively binding to miR-20a-5p, resulting in cell growth, metastasis, and apoptosis (Zhao et al., 2018). HOTAIR also promotes BC progression and metastasis by serving as a sponge for miR-129-5p to upregulate FZD7 expression (Wu et al., 2021). In a rat model of myocardial ischemia-reperfusion injury, HOTAIR upregulation promoted STAT3 expression, positively regulating the HOTAIR/miR-17-5p/STAT3 axis (Chen et al., 2021). In addition, HOTAIR also serves as ceRNA in multiple CNS disorders, such as Parkinson’s disease (PD) (Zhao et al., 2020), MS, and TBI (Duan et al., 2018). However, the specific role of miRNA expression regulation in the pathogenesis of CNS disorders remains to be further elaborated.

### Regulating Inflammatory Response

Sequencing analysis of the HOTAIR-related proteome showed that HOTAIR activates various proteins containing protein kinase domains and promotes the enrichment of important inflammatory signaling pathway proteins and their complexes, such as I-kappa B kinase complex, tumor necrosis factor alpha (TNF-α)/nuclear transcription factor-κB (NF-κB) signaling protein complex and the IKKα-IKKβ complex (Zhao et al., 2021). In addition, HOTAIR also upregulates the expression of multiple inflammatory signaling proteins, such as TNF-α and mitogen-activated protein kinase (Zhuang et al., 2015). For example, in ox-LDL induction in Raw264.7 cells (*in vitro* atherosclerosis model), HOTAIR overexpression reduced the inflammatory response and by NF-κB pathway by regulating FXR1. This indicated that HOTAIR may serve as a treatment target for preventing and treating atherosclerosis (Pang et al., 2018). HOTAIR knockdown reduced NF-κB target gene expression by inhibiting NF-κB and related cofactor recruitment at the target gene promoter in lipopolysaccharide-induced inflammatory response in macrophages (Obaid et al., 2018). Moreover, HOTAIR may regulate the gene transcription of key repressors of the NF-κB activation pathway through epigenetic regulation, thus participating in the immune escape of glioma cells (Wang et al., 2021). Therefore, HOTAIR expression may be involved in the development of CNS disorders by inhibition of inflammatory responses and release of inflammatory factors.

### Regulating Multiple Signaling Pathways

HOTAIR is involved in the regulation of multiple signaling pathways, especially in tumors. Guo et al. (2018) reported that upregulated HOTAIR expression activated the Wnt/β-catenin pathway, an important pathway related to tumor genesis and development. Further, it promoted cisplatin resistance *via* suppression-expression and alters intercellular signal transduction in lung cancer (Guo et al., 2018). In colorectal cancer (CRC), knockdown of HOTAIR inhibited the activation of Wnt/β-catenin pathway, resulting in inhibited proliferation and invasion of CRC cells and chemoresistance, suggesting that the HOTAIR/Wnt/β-catenin pathway may be a potential therapeutic target in CRC (Xiao et al., 2018).

Meanwhile, although the Wnt/β-catenin pathway was also found to be involved in the progression and, thus, the poor prognosis of glioma patients (Kaur et al., 2013), it is unknown whether HOTAIR plays a role in glioma pathogenesis by regulating the Wnt/β-catenin pathway. The phosphoinositide-3-kinase (PI3K)/AKT/mammalian target of rapamycin (mTOR) pathway plays a crucial role in the malignant transformation of human tumors and their subsequent proliferation and metastasis (Mabuchi et al., 2015). HOTAIR upregulation was identified in an ovarian cancer cell model, and it was found to increase tumor progression and promote tumor aggressiveness and metastasis *via* activation of the PI3K/AKT/mTOR pathway (Dong and Hui, 2016). Moreover, HOTAIR promoted the development of glioma and the expression of fibroblast growth factor 1 (FGF1), which promotes tumorigenesis by activating the PT3K/AKT pathway tumorigenic function (Hadari et al., 2001). However, aside from glioma, the role of HOTAIR in the pathogenesis, development, and prognosis of other CNS disorders through the regulation of more signaling pathways has not been clearly explained.

### Cell Cycle–Associated Gene Regulation

Studies have shown that HOTAIR knockdown in different tumor cells could affect EZH2-dependent cell cycle expression and induce cell cycle arrest (Kogo et al., 2011; Zhou et al., 2015). Gupta et al. (2010) was the first to report that in BC, two tumor suppressor genes (i.e., p21 and p16), which blocked cell cycle transition from G1 phase to S phase, are direct target genes of HOTAIR by chromatin immunoprecipitation. In glioblastoma studies, interference of HOTAIR expression in U87 and U87 VIII cells induced G1 phase arrest and increased expression of RB and dephosphorylated RB (Zhou et al., 2015). As RB is at the core of the G1 cell cycle regulation network, several growth factors (e.g., platelet-derived growth factor and epidermal growth factor) bind with their corresponding receptors to promote cyclin transcription. Activated cyclin and cyclin-dependent kinases (CDKs) form a complex to dephosphorylate RB, which can affect the transcription activity of the E2F family. The downstream transcription target genes of the E2F family, including B-Myb, c-Myc, and CDC2, are all essential proteins for the G1/S phase transition of cell cycle (Ertosun et al., 2016). The above results show that p16/P21 is activated after HOTAIR expression, and the regulatory network of cyclin D1→RB→E2F1 in tumor cells is blocked individually. Further, rapid passage of cell cycle from the G1 phase is eliminated, resulting in cell cycle arrest. Therefore, HOTAIR can be used as a candidate target for inhibiting the tumor cell cycle evolution. However, whether HOTAIR plays a role in CNS disorders by regulating cell cycle remains to be further determined.

### Regulating Protein Ubiquitination

HOTAIR acts as a platform for protein ubiquitination, helping assemble E3 ubiquitin ligases to bind to their respective substrates, promoting ubiquitination of the complex and accelerating its degradation. Mex3b and Dzip3 are E3 ubiquitin ligases with specific RNA-binding domains that bind HOTAIR. Mex3b exists in the nucleus and cytoplasm, and the corresponding ubiquitination substrate is Snurportin-1 protein. Dzip3, however, exists only in cytoplasmic vesicles and promotes the ubiquitination of Ataxin-1 protein (Yoon et al., 2013). Mex3b acts as E3 ubiquitin ligase in HOTAIR-induced degradation of Runt-related transcription factor 3 (Runx3), and silencing HOTAIR or Mex3b attenuates Runx3 degradation. Therefore, the interaction between HOTAIR and Mex3b can induce the ubiquitination of Runx3 protein and enhance the invasion ability of gastric cancer cells, and providing a potential therapeutic target for gastric cancer metastasis (Xue et al., 2018). HOTAIR binds to the androgen receptor (AR) protein to block its interaction with the E3 ubiquitin ligase murine double minute 2, thereby preventing AR ubiquitination and protein degradation and contributing to castration-resistant prostate cancer (Zhang et al., 2015). However, there are few studies on HOTAIR regulation of protein ubiquitination, and the role of HOTAIR in the ubiquitination process of different proteins in CNS disorders still needs to be further studied.

## HOTAIR Involvement in Pathogenesis of Central Nervous System Disorders

**FIGURE 2:**
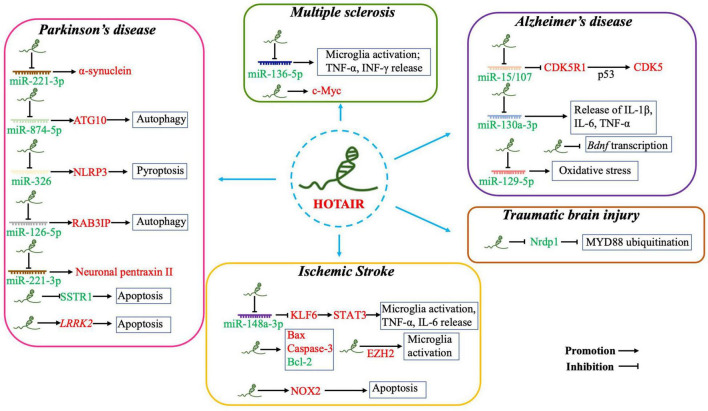
Overexpressed HOTAIR is involved in the pathogenesis of CNS disorders by sponging miRNAs, inducing apoptosis, promoting microglia activation and neuroinflammation, enhancing oxidative stress, and inhibiting *Bdnf* transcription. (i) Parkinson’s disease: HOTAIR acts as competing endogenous RNA by sponging miR-211-3p, miR-874-5p, miR-326, and miR-126-5p. HOTAIR also induces apoptosis by inhibiting SSTR1 or promoting LRRK2 expression. (ii) Ischemic stroke: Overexpressed HOTAIR acts as competing endogenous RNA by sponging miR-148a-3p to upregulate STAT3; promoting microglia activation and the release of pro-inflammatory factors; and upregulating EZH2, also a pathway regulated by HOTAIR to activate microglia. HOTAIR promotes apoptosis by upregulating Bax, Caspase-3, and NOX2, as well as by downregulating Bcl-2 expression. (iii) Alzheimer’s disease: Overexpressed HOTAIR acts as competing endogenous RNA by sponging miR-15/107, miR-130a-3p, and miR-129-5p to promote apoptosis, oxidative stress, and neuroinflammation. HOTAIR is also involved in the pathogenesis of AD by inhibiting *Bdnf* transcription. (iv) Multiple sclerosis: Overexpressed HOTAIR sponges miR-136-5p to promote microglia activation, release of TNF-α and IFN-γ, and upregulate c-Myc expression. (v) Traumatic brain injury: Overexpressed HOTAIR inhibits MYD88 ubiquitination to repress microglial activation. Red and green fonts represent high and low expression, respectively.

Dysregulated HOTAIR expression has been found in various pathological processes, including in CNS disorders (Figure 2). However, the specific mechanism of HORAIR involvement in the pathogenesis of CNS disorders is still controversial. HOTAIR may exert diverse functions in modulating pathological processes in different types of CNS disorders. In this part, we elucidated important evidence for understanding the crucial roles of the HOTAIR functional network in signaling pathways and identifying new diagnostic biomarkers, as well as therapeutic targets for CNS disorders (e.g., ischemic stroke, Alzheimer’s disease (AD), PD, MS, and TBI).

### HOTAIR in Ischemic Stroke

Stroke is one of the leading causes of death and disability worldwide, with ischemic stroke accounting for approximately 87% of all strokes (Bao et al., 2018). An ischemic stroke is mainly caused by a sudden stop of blood flow to the brain, which reduces the supply of oxygen and glucose to brain cells (Wang S. W. et al., 2018). Patients with ischemic stroke may present with varying neurological deficits, such as limb paralysis, language impairment, ataxia, and paresthesia (Balch et al., 2020). Diabetes, hypertension, high cholesterol, smoking, alcohol abuse, atrial fibrillation, and other factors are contribute to the risk of ischemic stroke (Koh and Park, 2017). However, current measures to protect the brain from ischemic stroke remain suboptimal (Satani and Savitz, 2016). Therefore, studies on the underlying mechanisms of ischemic brain injury are urgently needed.

HOTAIR expression is significantly elevated in permanent middle cerebral artery occlusion (pMCAO) mice, and this is accompanied with increased infarcted area, apoptosis of neural cells, and exacerbation of neurological deficits (Huang et al., 2021). Silencing HOTAIR expression reversed the above ischemic injury induced by cerebral artery occlusion, decreased pro-inflammatory factors (TNF-α and IL-6), and inhibited the apoptosis of neural cells. Increased HOTAIR expression was also observed in oxygen–glucose deprivation (OGD)-injured N2a cells. In addition, HOTAIR silencing markedly increased cell viability and reduced apoptosis rate and pro-inflammatory factors, indicating that HOTAIR suppression may exert protective roles in ischemic and hypoxic neural cells *via* anti-inflammation and anti-apoptosis. HOTAIR may also act as a ceRNA to impose posttranscriptional regulation (Liu et al., 2014).

miR-148a-3p was downregulated in OGD-injured N2a cells. In addition, as a target of HOTAIR, KLF6 is important in modulating inflammation and immune responses (Syafruddin et al., 2020). Upregulated KLF6 expression was found in ischemia/reperfusion (I/R) injury (Zhang Y. et al., 2020). Downregulated KLF6 expression promoted cell viability, decreased apoptosis, and repressed inflammatory response. HOTAIR silencing downregulated KLF6 expression by sponging miR-148a-3p; thus, modulating HOTAIR expression may protect I/R injured cells *via* the miR-148a-3p/KLF6 axis. KLF6 inhibited the expression of anti-inflammatory genes by suppressing STAT3 signaling (Chen et al., 2018). Activation of the STAT3 pathway promoted microglia/macrophage polarization toward an anti-inflammatory phenotype and ameliorated brain damage (Liu et al., 2019). HOTAIR silencing could activate the STAT3 pathway to protect against cerebral I/R injury and promote neurological recovery after ischemic stroke by downregulating KLF6 expression (Huang et al., 2021).

HOTAIR expression was also upregulated in an *in vitro* model of OGD/R injury. HOTAIR silencing reduced endothelial cell permeability and increased the expression of occludin, claudin-5, zonula occludens-1, and VE-cadherin in OGD/R-treated human brain microvascular endothelial cells. Moreover, HOTAIR knockdown significantly decreased the expression levels of the apoptosis-related proteins Bax and cleaved caspase-3 and significantly increased those of Bcl-2. Therefore, maintenance of the blood-brain barrier (BBB) structure and anti-apoptosis effects were the main cytoprotective mechanism of HOTAIR. EZH2 is a histone methyltransferase that has been shown to be involved in I/R injury (Jin D. et al., 2021). Inhibition of microglia activation and inflammatory response by EZH2 knockdown was one of the mechanisms by which it exerted its neuroprotective effect in hypoxic-ischemic brain injury (Xue et al., 2019). HOTAIR has been documented to be an EZH2-binding lncRNA, regulating EZH2 expression and recruiting EZH2 to MYC promoter sites (Wang Y. et al., 2018). EZH2 expression was significantly upregulated in OGD/R-treated cells, and EZH2 overexpression attenuated the effects of HOTAIR knockdown on cell viability, BBB permeability, and cell apoptosis. Wang et al. (2022a) reported that a positive association between HOTAIR and EZH2 expression could be an underlying mechanism in the pathology of I/R injury, and HOTAIR mediated OGD/R-induced cell injury in an EZH2-dependent manner (Wang et al., 2022a).

Similar conclusions were obtained by Yang et al., showing that HOTAIR expression was significantly increased in pMCAO mice. In addition, high HOTAIR expression promoted the onset of ischemic infarct and cell apoptosis, whereas HOTAIR silencing attenuated hypoxia-induced apoptosis of HT22 cells. Further study on the specific mechanisms suggested that the expression of nicotinamide adenine dinucleotide phosphate oxidase oxidase 2 (NOX2) might be involved in the apoptosis regulated by HOTAIR. NOX2, as a key part of the electron transport chain, plays an important role in the plasma membrane (Lambeth et al., 2000). NOX2 has been shown to be a major inducer of stroke, and deletion of the Nox2 gene significantly reduced infarct size after I/R treatment in mice (Kahles et al., 2007). In pMCAO mice, NOX2 expression was obviously increased, and NOX2 overexpression promoted HT22 cell apoptosis, but this was reversed by HOTAIR knockdown. Thus, it is speculated that HOTAIR may bind to NOX2, and HOTAIR silencing may be a treatment approach in ischemic stroke by downregulating NOX2 expression (Yang and Lu, 2016).

Altogether, these findings suggest that elevated HOTAIR expression may be involved in the pathogenesis of ischemic stroke, and silencing HOTAIR may play a therapeutic role in ischemic stroke through anti-inflammatory, anti-apoptotic, ceRNA, and other mechanisms. However, the exact mechanism of HOTAIR involvement in ischemic stroke needs to be further clarified to develop targeted therapies for ischemic stroke.

### HOTAIR in Alzheimer’s Disease

Alzheimer’s disease is the leading cause of dementia worldwide, inducing progressive impairment of cognitive function that seriously affects daily life (Liu, 2022). After age 65 years, the incidence of AD approximately doubles every 5 years, and 50% of the population aged ≥ 85 years has AD (Winblad et al., 2016). Currently, more than 30 million people worldwide have AD (Saez-Atienzar and Masliah, 2020). AD is often associated with other human pathologies making the treatment difficult (Surguchov, 2020). Multiple mechanisms are involved in the pathogenesis of AD, but it is characterized by two hallmark pathological features: β-amyloid (Aβ) plaque deposition and neurofibrillary tangle accumulation. However, because the etiology and pathogenesis of AD are yet to be fully elucidated, there is currently no effective treatment modality (Hou et al., 2019). Therefore, new therapeutic strategies need to be explored in both preclinical and clinical studies.

Cyclin-dependent kinase 5 (CDK5) is a proline-directed serine/threonine kinase involved in several developmental and physiological processes in the CNS (McLinden et al., 2012). Abnormal kinase activity is thought to play an important role in AD pathogenesis (Wang J. Z. et al., 2007). Particularly, CDK5 has been thought to exacerbate the development of two major pathological features of AD by inducing Aβ production and mediating Tau protein hyperphosphorylation (Liu S. L. et al., 2016). CDK5 activity depends on the activation of the p35 regulatory subunit and is related to its activation quantity. P35 is encoded by the cyclin-dependent kinase 5 regulatory subunit 1 (CDK5R1) gene, which has a highly conserved 3′ UTR. This indicates that post-transcriptional regulation plays a crucial role in controlling CDK5R1 expression (Zuccotti et al., 2014). miR-15/107 expression is downregulated, while CDK5R1 mRNA expression is upregulated in the hippocampus and cerebral cortex of AD patients, suggesting that miR-15/107 may be involved in the negative regulation of CDK5R1 expression (Moncini et al., 2017). lncRNAs may be provide another layer of complexity in the regulation of CDK5R1 expression associated with AD pathogenesis, a hypothesis proposed by Spreafico et al. (2018) In a HeLa cell model, the researchers identified that HOTAIR negatively regulated CDK5R1 expression through a positive action on miR-15/107. The result was also confirmed in AD patients, that is, HOTAIR is downregulated in the hippocampus and cerebellum. Therefore, upregulated HOTAIR expression may inhibit AD pathogenesis by regulating the p35/CDK5R1/CDK5 axis and reducing Aβ production and Tau protein hyperphosphorylation (Spreafico et al., 2018).

Regular physical exercise is confirmed to inhibit cognitive degeneration in AD patients (Lu et al., 2022). In APP/PS1 mice, voluntary exercise (VE) improves cognitive function evaluated using the Morris water maze test. In addition, VE reduced the level of HOTAIR in hippocampal tissues of APP/PS1 mice and helped inhibit inflammation. Conversely, HOTAIR overexpression counteracted the protective effect of VE on cognitive function and promoted the release of inflammatory factors (IL-1β, IL-6, and TNF-α), suggesting that HOTAIR may promote AD by promoting inflammatory response. Therefore, the HOTAIR/miR-130a-3p axis may be involved in the pathological mechanism of AD through the inflammatory response, and thus, inhibiting HOTAIR expression may be a feasible therapeutic strategy for AD. Further exploration of the mechanisms by which HOTAIR regulates the inflammatory response *via* miR-130a-3p sponging is needed.

Inhibition of HOTAIR expression also rescued sevoflurane-mediated brain function impairment in rats. Sevoflurane induces neuronal apoptosis, and the resulting neurotoxicity is thought to be one of the causes of cognitive impairment and AD in elderly patients (Brosnan and Bickler, 2013). A possible mechanism by which sevoflurane causes cognitive impairment is through impaired transcription of mRNA encoding brain-derived neurotrophic factor (*Bdnf*) (Stiegler and Tung, 2014). *Bdnf* mRNA levels in the hippocampus of rats were significantly reduced after sevoflurane treatment. In the study by Wang et al., decreased *Bdnf* expression after sevoflurane treatment was mediated by HOTAIR lncRNA, as indicated by *Bdnf* expression coming close to normal after siRNA intervention for HOTAIR. Thus, HOTAIR inhibition may exert a role in the treatment of AD through upregulation of *Bdnf* transcription (Wang J. Y. et al., 2018). Similar conclusions were found by Wang et al.; in their isoflurane (ISO)-evoked HT22 cell model, downregulated HOTAIR expression contributed to the recovery from abnormal viability, apoptosis, inflammation, and oxidative stress through miR-129-5p regulation. miR-129-5p as a ceRNA of HOTAIR mediated its impact on cognition and oxidative stress in the ISO-injured SD rat model (Wang et al., 2022b).

Collectively, *in vitro* and *in vivo* studies reported that HOTAIR was highly expressed in AD models. Inhibition of HOTAIR expression may exert anti-AD effects through various mechanisms, including anti-inflammatory response, anti-apoptosis, and oxidative stress. In-depth studies on the mechanism of HOTAIR in the pathogenesis of AD are needed to develop therapeutic strategies for AD.

### HOTAIR in Parkinson’s Disease

As the second most common neurodegenerative disease after AD, PD has a high incidence rate (Spatola and Wider, 2014). In Europe, the prevalence and incidence rates of PD are approximately 108-257/100 000 and 11-19/100 000 per year, respectively (Balestrino and Schapira, 2020). Further, PD is prevalent in 1.37% of people aged over 60 years. In China, the incidence of PD is as high as 3.62 million (Qi et al., 2021). With the aging of the population, the incidence of PD is expected to double in the next 20 years (Chen, 2010). PD is characterized by static tremor, bradykinesia, stiffness, and other symptoms that reduce quality of life, culminating in severe disability due to inability to control motor function (Ma C. L. et al., 2014). PD is mainly caused by the degeneration and loss of dopaminergic neurons in the substantia nigra and the significant decrease of dopamine content in the striatum (Zhang W. S. et al., 2021). Studies have shown that the pathogenesis of PD includes α-synuclein misfolding and aggregation, mitochondrial dysfunction, protein clearance disorder, neuroinflammation, and oxidative stress (Jankovic and Tan, 2020). In addition, genetic factors such as mutations in genes encoding leucine-rich repeat kinase 2 (LRRK2) and GBA are common genetic risk factors for familial and sporadic PD (Riboldi and Di Fonzo, 2019; Jeong and Lee, 2020).

The complex etiology of PD poses an urgent need to identify a useful therapeutic target (Surguchov, 2022). HOTAIR was highly expressed in *in vitro* and *in vivo* models of PD, and downregulated HOTAIR increased cell viability and reduced cell apoptosis and inhibited the secretion of inflammatory cytokines and oxidative stress reaction. As a presynaptic neuronal protein, α-synuclein is a key factor in the pathogenesis of PD (Shahnawaz et al., 2020). Prevention of α-synuclein aggregation and reduction of its neurotoxicity could be a potential approach to improve the pathogenesis of PD (Fayyad et al., 2019). Sun et al. (2022) found a HOTAIR/miR-221-3p/α-synuclein signaling axis in a 1-methyl-4-phenyl-1,2,3,6-tetrahydropyridine-hydrochloride (MPTP)-induced mouse model of PD. Further, miR-221-3p can directly bind to HOTAIR and α-synuclein. As a ceRNA, HOTAIR regulates α-synuclein expression by sponging miR-221-3p (Sun et al., 2022). Therefore, overexpressed HOTAIR may be a pathogenic factor of PD, and HOTAIR inhibition reduced α-synuclein aggregation and toxicity in PD models, suggesting the potential therapeutic value of HOTAIR in PD.

Similarly, high HOTAIR expression was also observed in MPP^+^-induced SK-N-SH cell models. Impairment of autophagy has been reported to be involved in the pathogenesis of PD (Lu et al., 2020). Autophagy initiation requires the expression of autophagy-related 10 (ATG10) (Song et al., 2017). Zhao et al. (2020) identified a regulatory axis in HOTAIR/miR-874-5p/ATG10, and highly expressed HOTAIR increased ATG10 expression by sponging miR-874-5p, indicating the HOTAIR was involved in PD by enhancing autophagy. miR-326 has been reported to suppress inducible nitric oxide synthase expression and promote autophagy of dopaminergic neurons (Zhao et al., 2019) in PD. In addition, it was also a target of HOTAIR. In a mice model of PD, HOTAIR silencing repressed neuronal damage by inhibiting NOD-like receptor family pyrin domain-containing protein 3-mediated pyroptosis activation *via* regulation of miR-326 (Zhang Q. et al., 2021).

The *Rab3a* interacting protein (RAB3IP) regulates neurite growth and spinal cord development, suggesting that RAB3IP plays a regulatory role in neurons (Ren et al., 2018). RAB3IP has been confirmed to inhibit the autophagy of mammalian cells through its non-conservative C-terminal region, and autophagy impairment leads to the loss of age-related dopaminergic neurons, corresponding to the loss of dopamine in the striatal body and the accumulation of α-synuclein (Amagai et al., 2015). Lin et al. (2019) found that RAB3IP expression was upregulated in a PD mouse model, and downregulated RAB3IP expression suppressed autophagy and cell apoptosis. They further suggested that HOTAIR knockdown inhibited RAB3IP expression by acting as a ceRNA for miR-126-5p (Lin et al., 2019). Furthermore, HOTAIR downregulation also potentially inhibited the autophagy of dopaminergic neurons in the substantia nigra compacta. Mechanistically, HOTAIR can affect the expression of neuronal pentraxin II by sponging miR-221-3p (Lang et al., 2020). Therefore, HOTAIR may serve as a potential therapeutic target in PD.

High HOTAIR expression can accelerate the progression of dyskinesia in PD. In a PD cell model, HOTAIR bound to the promoter region of somatostatin receptor 1 (SSTR1), leading to increased methylation of SSTR1 by recruiting DNA methyltransferases. Notably, in a MPTP-induced PD mouse model, overexpressed HOTAIR stimulated DNA methylation of SSTR1 to reduce SSTR1 expression, thereby accelerating dyskinesia and facilitating dopaminergic neuron apoptosis (Cai et al., 2020). *LRRK2* gene mutations have been widely recognized as the most common cause of dominantly inherited PD and one of the risk factors for sporadic PD (Tolosa et al., 2020). Several studies demonstrated that repression of LRRK2 kinase activity was a potential therapeutic strategy for the treatment of PD (Blanca Ramírez et al., 2017). A regulatory relationship between HOTAIR and *LRRK2* mRNA was observed in a PD cell model, and Liu S. et al. (2016) found HOTAIR overexpression increased the stability of *LRRK2* mRNA and upregulated its expression in MPP^+^-induced SH-SY5Y. In addition, HOTAIR knockdown provided protection against MPP^+^-induced dopamine neuronal apoptosis by suppressing caspase 3 activity (Wang S. et al., 2017).

In summary, *in vivo* and *in vitro* models of PD showed enhanced expression of HOTAIR, and high HOTAIR expression promoted the occurrence and development of PD. Inhibited HOTAIR expression mainly exerted a therapeutic effect on PD by regulating the expression of target miRNAs and downstream genes at the post-transcriptional level. The possible neuroprotective mechanisms included anti-inflammatory, anti-oxidant stress, inhibition of autophagy, and anti-apoptotic effects. Moreover, the direct regulation of key pathogenic genes of PD (*LRRK2*) was also one of the important mechanisms by which HOTAIR was involved in the pathogenesis of PD. Therefore, inhibition of HOTAIR levels may be an effective disease-modifying strategy in PD.

### HOTAIR in Multiple Sclerosis

Multiple sclerosis (MS) is an autoimmune disease with an increasing incidence worldwide (Browne et al., 2014). Complex gene-environment interactions play an important role in the pathogenesis of MS, but the underlying causes and mechanisms behind the increase in MS incidence remain unclear (Lemus et al., 2018). The current results suggest that adverse lifestyle and changes in the human body environment can lead to the activation of peripheral immune cells. These cells secrete inflammatory factors to infiltrate the CNS, leading to glial proliferation, myelin demyelination, and secondary axonal injury (Dobson and Giovannoni, 2019). Epidemiological studies on MS suggest that low serum vitamin D (VD) level, smoking, childhood obesity, and Epstein-Barr virus infection may be involved in the occurrence of the disease (Wang et al., 2020). Clinically, MS is primarily manifested as sensory disturbances, autonomic nervous dysfunction, and ataxia (Kamińska et al., 2017).

Clinical studies have shown that microglia activation is associated with disease severity in MS patients (Rothhammer et al., 2018). Microglia is directly involved in cuprizone-induced demyelination by producing pro-inflammatory cytokines including TNF-α and interferon-γ (Klein et al., 2018). In cuprizone-induced demyelination, HOTAIR expression was upregulated, and this promoted microglia transformation into a pro-inflammatory M1-like phenotype and release of pro-inflammatory factors by regulation of miR-136-5p expression. Duan et al. (2018) proved that sulfasalazine inhibited M1 polarization of microglia and release of inflammatory factors by inhibiting HOTAIR expression, promoting myelin regeneration. This suggests that inhibition of HOTAIR expression is a potential approach in the treatment of MS (Duan et al., 2018).

MS is a complex disease that may be caused by the interaction between genetic and environmental risk factors, with VD deficiency considered to be the main cause (Hernández-Ledesma et al., 2021). Although the molecular mechanisms of VD protection in MS are not fully understood, the immunomodulatory properties of VD may be crucial in mediating its effects. For example, VD supplementation in patients with MS has been shown to regulate cytokine production and T-cell proliferation (Shirvani-Farsani et al., 2017). VD has also been reported to regulate HOTAIR expression (Jiang and Bikle, 2014; Riege et al., 2017). The exact mechanism by which VD regulates HOTAIR expression in MS remains unclear. However, a possible mediator is the c-Myc factor, which has been shown to regulate the effects of VD (Piek et al., 2010) and activate HOTAIR expression (Ma M. Z. et al., 2014). Pahlevan Kakhki et al. (2018) found that HOTAIR expression was dysregulated in both MS patients and animal models and was correlated with immune-activated sites. The role of HOTAIR in the pathogenesis of MS is still unclear, but it is speculated that HOTAIR might play a role in CNS-related processes (e.g., myelination) through various epigenetic mechanisms, including histone modification, chromatin remodeling, and miRNA regulation. These speculated mechanisms are consistent with the known immunomodulatory and neuroprotective effects of VD (Pahlevan Kakhki et al., 2018).

### HOTAIR in Traumatic Brain Injury

Traumatic brain injury is characterized by high disability and mortality; however, there is currently no effective treatment modality for TBI (Stein et al., 2010). It can usually be divided into primary and secondary injuries (Najem et al., 2018). Neuroinflammation is a hallmark of TBI and includes activation of resident glial cells (microglia and astrocytes), recruitment of immune cells, and release of inflammatory mediators in the brain (Simon et al., 2017). Abnormal activation of microglia and excessive release of inflammatory factors play an important role in secondary injury of TBI, and these mechanisms hinder nerve injury repair post-TBI (Lee et al., 2019). Many studies have shown that inhibition of TBI-induced microglial activation and neuroinflammatory response is beneficial for improving TBI (Corrigan et al., 2016; Kumar et al., 2017). Therefore, it is important to explore strategies to alleviate TBI by inhibiting microglial activation and inflammatory factor release.

HOTAIR is significantly upregulated in activated microglia of TBI mice, and silencing HOTAIR could inhibit microglial activation and reduce the release of IL-1β, IL-6, and TNF-α. Cheng et al. (2021) further explored the regulatory mechanism of HOTAIR on microglia activity and found that HOTAIR may directly inhibit microglia activation by binding myeloid differentiation factor-88 adaptor protein (MYD88). MYD88 is a crucial linker molecule in the toll-like receptor signaling pathway, and its expression is significantly upregulated in TBI mice. Downregulated MYD88 expression can improve TBI by reducing microglial activation (Zhu et al., 2014; Leite et al., 2015). Nrdp1, a member of the E3 ubiquitin ligase family, can downregulate the protein level of MYD88 by mediating MYD88 ubiquitination (Wang et al., 2009). Cheng et al. (2021) explored the effect of HOTAIR on microglia activation in TBI mice and found that overexpression of HOTAIR stabilized MYD88 by inhibiting Nrdp1-mediated MYD88 ubiquitination, preventing microglia overactivation and inflammatory factor release after TBI and improving prognosis (Cheng et al., 2021).

## Diagnostic and Prognostic Significance of HOTAIR in Central Nervous System Disorders

Early diagnosis is crucial for the treatment and prognosis of CNS disorders, but current diagnostic methods are inadequate for early diagnosis of CNS disorders. Therefore, it is important to improve diagnostic methods and develop highly specific and sensitivity biomarkers. lncRNA is expressed in various tissues; although the expression level is lower than that of protein-coding genes, its tissue specificity is much higher (Pan et al., 2018). This gives it potential advantages as a biological marker with highly specific diagnostic functions. Abnormal expression of lncRNAs not only exist in tissues and cells, but also in various body fluids, including blood, urine, saliva, and gastric juice (Sartori and Chan, 2014). In addition, lncRNAs remain stable in circulating plasma even in the presence of ribonuclease (Ren et al., 2013). However, the mechanism by which circulating lncRNAs are secreted into the blood and stably exists has not been fully elucidated. Exosomes can better protect plasma lncRNAs against the degradation of ribonucleases. Under pathological conditions, cells can selectively load lncRNAs into exosomes, resulting in high specificity for disease diagnosis (Gezer et al., 2014).

HOTAIR has been reported to contribute to the pathogenesis of MS both in animal models and in human studies. In addition, it might also be a risk locus of MS. Taheri et al. (2020) genotyped three single-nucleotide polymorphisms (SNPs) in HOTAIR, namely, rs12826786, rs1899663, and rs4759314, in Iranian MS patients and healthy subjects and found that rs4759314 SNP was associated with the risk of MS in an allelic model [OR (95% CI) = 1.34 (1.08–1.67)]. This suggested that HOTAIR might be a risk locus for MS in the Iranian population (Taheri et al., 2020). Soltanmoradi et al. (2021) enrolled 60 RRMS patients (30 patients with relapse, 30 patients with remission) to evaluate the diagnostic value of HOTAIR in MS patients. The results showed higher levels of HOTAIR in relapse patients than in remission patients. Moreover, there was a positive correlation between high HOTAIR expression and increased levels of matrix metalloproteinases 9 in peripheral blood mononuclear cells of MS patients with relapse. Further, the receiver operating characteristic analysis showed the potential of HOTAIR (area under the curve: 0.87; sensitivity: 83%; specificity: 80%) as biomarkers for distinguishing between relapse and remission phases of RRMS (Soltanmoradi et al., 2021).

Currently, there are only few studies on HOTAIR as a diagnostic marker of CNS disorders, and these studies remain at the level of HOTAIR expression. Research on its regulation mechanism is limited, and thus, HOTAIR cannot be directly applied in clinical practice. However, with the development of body fluid detection, in the context of precision medicine, lncRNA measurement has become a conducive method for accurate diagnosis and development of individualized prevention and treatment plans (Kumar and Goyal, 2017; He et al., 2021). Compared with traditional diagnostic markers of CNS disorders, lncRNA has higher specificity and sensitivity (Chandra Gupta and Nandan Tripathi, 2017), and thus, it is expected to become a new biological indicator for the diagnosis of CNS disorders.

## Conclusion

In conclusion, recent studies have shown that HOTAIR is involved in the occurrence and development of several CNS disorders and may be a potential biomarker for disease diagnosis and prognosis. *In vitro* and *in vivo* studies have shown that inhibition of HOTAIR expression can play a therapeutic role in CNS disorders through multiple mechanisms, suggesting it may be a potential therapeutic target for CNS disorders.

## Author Contributions

JW and JZ wrote the manuscript. PH, LG, and ST produced the figures. ZH edited and revised the review. All authors have read and approved the final manuscript.

## Conflict of Interest

The authors declare that the research was conducted in the absence of any commercial or financial relationships that could be construed as a potential conflict of interest.

## Publisher’s Note

All claims expressed in this article are solely those of the authors and do not necessarily represent those of their affiliated organizations, or those of the publisher, the editors and the reviewers. Any product that may be evaluated in this article, or claim that may be made by its manufacturer, is not guaranteed or endorsed by the publisher.
